# Rapid Purification of Recombinant Histones

**DOI:** 10.1371/journal.pone.0104029

**Published:** 2014-08-04

**Authors:** Henrike Klinker, Caroline Haas, Nadine Harrer, Peter B. Becker, Felix Mueller-Planitz

**Affiliations:** 1 Molecular Biology Unit, Adolf-Butenandt-Institute, Ludwig-Maximilians-Universität München, Munich, Germany; 2 Center for Integrated Protein Science Munich, Germany; 3 Department of Biochemistry, Ludwig-Maximilians-Universität München, Munich, Germany; 4 Gene Center, Ludwig-Maximilians-Universität München, Munich, Germany; The National Institute of Diabetes and Digestive and Kidney Diseases, United States of America

## Abstract

The development of methods to assemble nucleosomes from recombinant histones decades ago has transformed chromatin research. Nevertheless, nucleosome reconstitution remains time consuming to this day, not least because the four individual histones must be purified first. Here, we present a streamlined purification protocol of recombinant histones from bacteria. We termed this method “rapid histone purification” (RHP) as it circumvents isolation of inclusion bodies and thereby cuts out the most time-consuming step of traditional purification protocols. Instead of inclusion body isolation, whole cell extracts are prepared under strongly denaturing conditions that directly solubilize inclusion bodies. By ion exchange chromatography, the histones are purified from the extracts. The protocol has been successfully applied to all four canonical *Drosophila* and human histones. RHP histones and histones that were purified from isolated inclusion bodies had similar purities. The different purification strategies also did not impact the quality of octamers reconstituted from these histones. We expect that the RHP protocol can be readily applied to the purification of canonical histones from other species as well as the numerous histone variants.

## Introduction

The development of a method to reconstitute nucleosomes from recombinant histone proteins and DNA constituted a milestone in chromatin research [1]–[3]. Current research still heavily depends on the availability of sufficient quantities of pure and homogenous nucleosomes that can, for example, be used as substrates for histone modifying enzymes, to characterize the interactions of nucleosome binding factors, to generate nucleosome arrays for physicochemical analysis, or to explore the function of the many naturally occurring histone variants.

**Figure 1:**
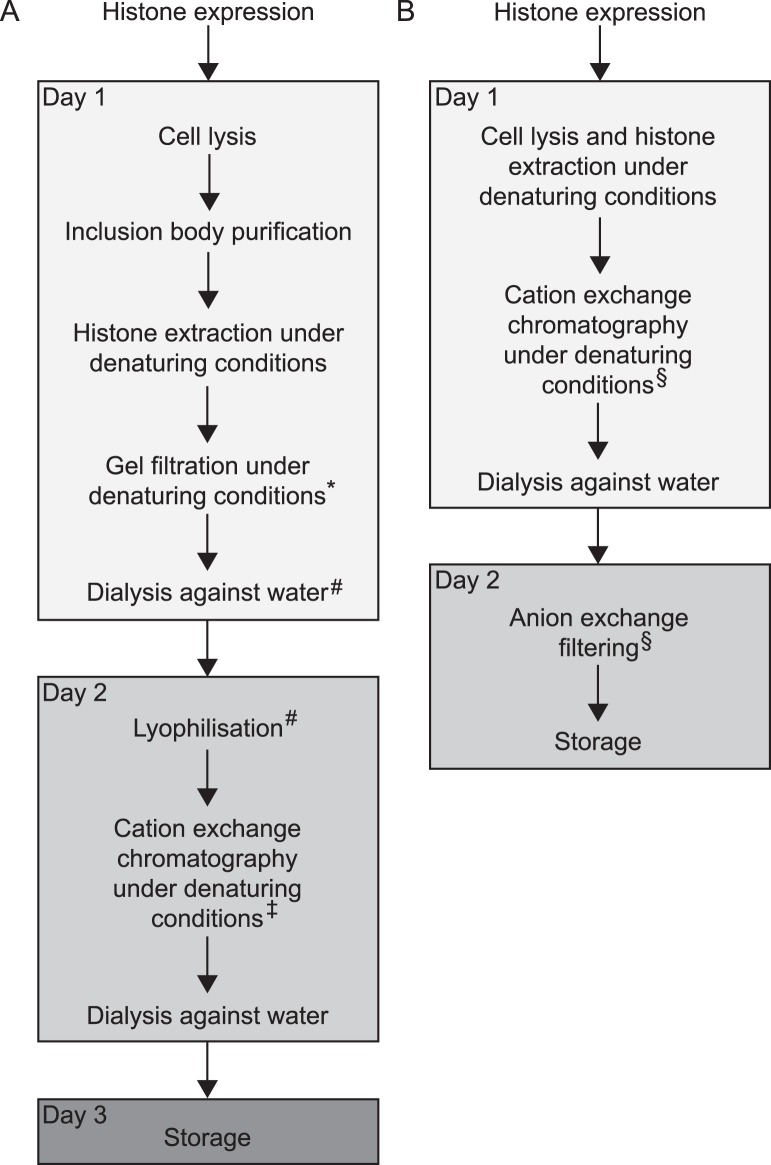
Histone purification strategies. Schematic depiction of the workflow of (A) the conventional histone purification method according to Luger and coworkers [2], [3] and (B) our RHP protocol. For further details see the main text. Footnotes indicate variations and simplifications of the initial protocol. * The gel filtration step was successfully omitted in simplified purification schemes [5], [7]–[9]. # These steps can be replaced by dilution into or dialysis against SAU 200 buffer [5]–[9]. ‡ To remove possible DNA contaminations, it was suggested to filter the sample through an anion exchange resin prior to applying it to the cation exchange chromatography [7]–[9]. § Anion exchange filtering and cation exchange chromatography can be combined. See note in step 3.2 and Figure S2 in File S1.

Recombinant histones are commonly expressed in bacteria where they typically partition into inclusion bodies [4]. Therefore, standard protocols begin with the preparation of inclusion bodies, which are isolated from the insoluble fraction of whole cell extracts in a series of washing steps [1]–[3]. During each washing step, the insoluble fraction is resuspended in buffer and then pelleted again by centrifugation. To stringently remove impurities, detergent is added to the buffer during the first washing steps. Subsequent washes serve to dilute the detergent. Next, the histones are solubilised and extracted from the inclusion bodies under denaturing conditions by addition of DMSO followed by incubation in a buffer containing 7 M guanidine hydrochloride. Further purification of the histones is achieved by gel filtration and cation exchange chromatography in a urea-based buffer. A final dialysis against water is required to remove salt and urea (Fig. 1A).

The purification of recombinant histones, however, is time-consuming and often rate-limiting for many applications. Several short-cuts to the original purification method were suggested to speed up the procedure. For example, the gel filtration and a lyophilisation step preceding the cation exchange chromatography have been successfully omitted [5]–[9] (Fig. 1A). Nevertheless, the purification of the inclusion bodies through the series of long centrifugation and laborious resuspension procedures remained the bottle-neck of the purification procedure. Even worse, we observed that preparation of inclusion bodies can lead to loss of material for some histones, in particular *Drosophila* H2B (Fig. S1 in File S1). A radically different way to obtain histone octamers was taken by co-expression of all four histones in bacteria. *In vivo*, the histones formed octamers, which could be isolated under native conditions [10]. It may be preferable, however, to individually purify the histones for several applications, for instance to incorporate specifically modified histones, so called designer histones, into octamers.

Here, we introduce a simplified and robust protocol, termed RHP, for the purification of individual histones expressed in bacteria that circumvents the laborious isolation of the inclusion bodies (Fig. 1B). The method uses denaturing conditions already during cell lysis to extract the histones. Therefore, this strategy is also applicable to histone derivatives that do not fully partition into inclusion bodies. Similar to standard methods, the histones are purified by cation exchange chromatography. To avoid DNA contaminations, we recommend filtering the solution through an anion exchange resin as suggested previously [7]–[9]. The RHP method requires considerably less hands-on working time than previous methods. Histones purified according to the RHP method readily formed histone octamers, and these octamers were comparable in purity to octamers that were reconstituted from more traditional histone preparations [5]. Octamers that contained RHP histones have already been successfully used in a number of previous studies [11]–[13].

## Materials and Methods

The protein content of samples taken throughout the purification procedure was analyzed on 15 or 18% SDS gels by Coomassie staining. The gels were scanned with the Odyssey Infrared Imaging System (LI-COR).

### Histone expression

*Drosophila* histones H3 and H4 were expressed from pET3c-based constructs [14]. Codon-optimized genes for *Drosophila* H2A and H2B were synthesized and subcloned into pET15b (pFMP128 and pFMP129, respectively; Table S1 in File S1; Eurofins MWG). BL21(DE3) cells were transformed with the expression plasmids and grown at 37°C to a density of OD_600_ 0.6–0.8 in LB supplemented with Ampicillin (100 mg/l) in shaking cultures (1.3 to 4 l). Histone expression was induced by addition of 1 mM IPTG. After 2 h, the cells were harvested by centrifugation at 4°C and stored at −80°C.

Histone expression was verified by removing 1 ml of the culture directly before induction and before harvesting. Cells in these samples were pelleted, resuspended in sample buffer (150 µl per OD_600_; 9 M urea, 1% SDS, 25 mM Tris-Cl pH 6.8, 1 mM EDTA, 0.02% Bromophenol Blue, 100 mM DTT) and heated (15 min at 65°C). It is recommended to strongly vortex the whole cell extract to shear genomic DNA. The protein contents of equivalent amounts of the extracts were analyzed on SDS gels.

### Cell lysis and histone extraction

The bacteria pellet was resuspended in SAU buffer (40 mM NaOAc pH 5.2, ≥6 M urea, 1 mM EDTA pH 8, 5 mM β-Mercaptoethanol, 10 mM lysine) supplemented with 200 mM NaCl (SAU 200) and protease inhibitors (1 mM PMSF, 1 mg/l Aprotinin, 1 mg/l Leupeptin, 0.7 mg/l Pepstatin). We typically resuspend the cell pellet of up to 6 l cultures in a final volume of 35 ml. Defined buffer conditions are most conveniently achieved by adding 10x SA buffer (400 mM NaOAc pH 5.2, 10 mM EDTA pH 8, 100 mM lysine), β-Mercaptoethanol and NaCl to a final concentration of 5 mM and 200 mM, respectively, and protease inhibitors directly to the cell pellet. Once the cells are properly resuspended, urea powder is added to a concentration of 6 M and the suspension is filled up to the final volume with water.

All steps during lysis and purification were performed at 4°C. Cells were lyzed by three passes through a French Press (1,500 psi; Thermo Spectronic) and sonication on ice (at least 2 min effective sonication time with an amplitude of 30% with pulses of 15 sec followed by 30 sec pauses; Branson Ultrasonics). If urea is added as powder as described above, we recommend to perform sonication prior to the French Press and to use longer sonication times (up to 20 min effective sonication time with occasional mixing) to ensure that all urea is fully dissolved.

The extract was cleared by centrifugation for 20–30 min at ∼41,000 g (SS-34 rotor, Sorvall RC 6 Plus; Thermo Scientific) and filtration. For the initial histone preparations, conventional 0.45 µM syringe filters were used. These filters easily clogged in contrast to syringe filters containing a glass-fiber prefilter (HPF Millex, Millipore) that were successfully employed in later preparations.

### Cation and anion exchange

With the exception of one purification shown in Figure S2 in File S1 (variant 2), the pre-cleared cell extract was directly passed over a HiTrap SP HP column (5 ml; GE Healthcare) that was pre-equilibrated in SAU 200 buffer. In variant 2 of the RHP method shown in Figure S2 in File S1, the extract was applied onto a HiTrap Q HP column (GE Healthcare) that was stacked on top of a SP column (both 5 ml), such that the flow-through of the Q column directly ran into the SP column. When the extract had passed completely through the Q column, the Q column was removed from the FPLC system. The washing and elution steps were then carried out only with the SP column as described next.

The SP column was washed with 200 to 300 mM NaCl for several column volumes (CV). Histones were eluted with a NaCl gradient. Pooled histone-containing fractions were dialyzed against cold water over night (3 times ≥3 l) in dialysis tubing with a molecular weight cut-off of 6000–8000 Da (Spectra/Por).

After dialysis, the sample was passed over a Q HP column (1 or 5 ml; pre-equilibrated in 15 mM Tris-Cl pH 8) unless indicated otherwise (Figure S2 in File S1, variant 2). To this end, the dialysate was centrifuged first to remove precipitates, then supplemented with 15 mM Tris-Cl pH 8 and filtered using conventional syringe filters (0.45 µm). The flow-through of the column was collected. To regenerate the resin and to control whether a fraction of the histones had bound to the resin, a gradient up to 2 M NaCl was applied. The elution fractions generally contained negligible amounts of H2A, H2B or H4. A fraction of H3 (less than a quarter) sporadically bound to the resin. This fraction was not analyzed further.

Histone concentrations were determined by absorption measurement at 280 nm (see Table 1 for the extinction coefficients). Yields obtained with the RHP method typically ranged from 2 to 15 mg per liter expression culture and were comparable within day-to-day variances to purifications from inclusion bodies. Purities were calculated according to ref. [15]. Aliquots of the purified histone were flash frozen in liquid nitrogen.

**Table 1:** Extinction coefficients of *Drosophila* histones at 280 nm.

	Molecular weight (Da)*	ε_280_ (cm^−1^ M^−1^)#
**H2A**	13,232	4,470
**H2B**	13,565	7,450
**H3**	15,257	4,470
**H4**	11,250	5,960
**Octamer**	106,608	44,700

*Molecular weights do not include the initial methionine.

# The extinction coefficients were calculated using the ProtParam tool with water as solvent (Swiss Institute of Bioinformatics; http://web.expasy.org/protparam/) [17].

#### Purification of histones from inclusion bodies

Preparation of recombinant histones from purified inclusion bodies was done essentially as described [5]. In short, BL21(DE3) cells that expressed histones were lysed in 50 mM Tris-Cl pH 7.5, 100 mM NaCl, 1 mM EDTA pH 8, 5 mM β-Mercaptoethanol in the presence of protease inhibitors by sonication and French Press as described above. Inclusion bodies were purified by a succession of four washing steps using lysis buffer that was supplemented with Triton X-100 (1%) during the first two washes. Histones were extracted from inclusion bodies by homogenization in DMSO and unfolding buffer (7 M guanidine hydrochloride, 20 mM Tris-Cl pH 7.5, 10 mM DTT). After dialysis against SAU 200 buffer, cation exchange chromatography and subsequent dialysis against water were performed as described above.

#### Octamer assembly

Histone octamers were assembled with ∼1 mg of each *Drosophila* histone according to Luger and coworkers [2], [3]. Histones were lyophilized (Alpha 1–2, Christ; RZ 2.5, vacuubrand) and solubilized in unfolding buffer as described in the Results and Discussion section (see step 6.2). To analyze histone stoichiometry by SDS-PAGE, the samples were diluted in water (1∶10) prior to loading to reduce the concentration of guanidine hydrochloride, which can negatively affect the gel run. Dialysis into refolding buffer (3 times 2 l; 10 mM Tris-Cl pH 7.5, 2 M NaCl, 1 mM EDTA, 5 mM β-Mercaptoethanol) was performed in dialysis tubing with a molecular weight cut-off of 6000–8000 Da. Precipitates were removed by centrifugation and the sample was loaded onto a size exclusion chromatography column (Superdex 200 HiLoad 16/60, 120 ml; GE Healthcare) pre-equilibrated in refolding buffer. Elution fractions were analyzed by SDS-PAGE. Octamer-containing fractions were pooled according to purity and histone stoichiometry. After concentration to 2–3 mg/ml in centrifugal filters (Amicon Ultra-4 or Microcon, 30 kDa MWCO; Millipore), the octamers were stored as described in the Results and Discussion section. Yields were (19±10)% (n = 3).

## Results and Discussion

Here we present the RHP method for the purification of recombinant histones from bacteria. With this method, we purified the four canonical histones from *Drosophila melanogaster*. Human histones, the *Drosophila* histone variant H2Av as well as several histone H3 and H4 mutants including tail-deleted H4 can be purified using the same protocol (Jens Michaelis, personal communication, and data not shown). For clarity, the important steps of the RHP protocol are listed as bullet points. Further details are given in the Materials and Methods section.

### 1 Histone expression

Some histones express poorly in bacteria. Species bias of codon usage of the recombinant gene or toxicity of the gene product are two potential causes of poor expression. *Drosophila* H2A and H2B, for example, showed low and variable expression levels, whereas H3 and H4 always expressed robustly from the same vector (pET3c). To circumvent codon bias and toxicity, we codon-optimized the genes for H2A and H2B (Table S1 in File S1) and cloned them into a vector that provides a more stringent control over the expression through co-expression of the *lac* repressor (pET15b). H2A and H2B were more robustly expressed from these optimized expression plasmids in standard BL21(DE3) *E. coli* cells. Histone expression comprised the following steps:

#### 1.1 Transformation of BL21 (DE3) *E. coli* with the respective expression plasmid

Depending on the expression plasmid and the source of the histones, bacterial strains expressing rare tRNAs or strains that restrict leaky expression may improve the yield.

#### 1.2 Growth of up to 6 L of culture to OD_600_ = 0.6–0.8

#### 1.3 Induction of histone expression by addition of 1 mM IPTG for 2 h at 37°C

We recommend verifying the expression by SDS-PAGE before proceeding with the protocol (see Materials and Methods). Overexpression must be clearly visible in whole cell extracts.

#### 1.4 Harvesting of the cells by centrifugation

After centrifugation, it is recommended to resuspend the cells in small volumes of cold water, transfer the cells to a 50 ml conical tube and pellet the cells a second time.

#### 1.5 Storage of the bacteria pellets at −80°C until further use

### 2 Cell lysis and histone extraction

Histones expressed in bacteria are typically insoluble and form inclusion bodies. To extract both the soluble and insoluble fraction, whole cell extracts were prepared under denaturing conditions in presence of ≥6 M urea. Addition of free lysine to the lysis buffer served to prevent carbamylation of the histone proteins that might occur upon reaction with natural degradation products of urea [16]. Nevertheless, exposure of the histone proteins to the urea-containing buffer (SAU buffer) should be kept to a minimum. Also note that the urea solution should always be prepared freshly and never be warmed up. Cell lysis and histone extraction required the following steps:

#### 2.1 Resuspension of the bacteria pellet in sodium-acetate-urea (SAU) buffer containing 200 mM NaCl (SAU 200)

Care should be taken that the final urea concentration during cell lysis and extraction is between 6–7.5 M to assure efficient protein denaturation and histone extraction without exceeding the solubility of urea. A defined final urea concentration is most conveniently achieved by resuspending the cell pellet in an appropriate volume of 10x SA buffer and adding urea powder directly to the suspension. Water is added and the suspension is mixed to dissolve the urea.

#### 2.2 Cell lysis by French Press and sonication

The order of the lysis steps is optional. However, we recommend to sonicate the sample before employing the French Press. This order of events has the advantage that urea has additional time to dissolve during sonication so that it does not clog the French Press. Note that the French Press step may be left out completely, as it only marginally enhanced extraction (data not shown).

**Figure 2:**
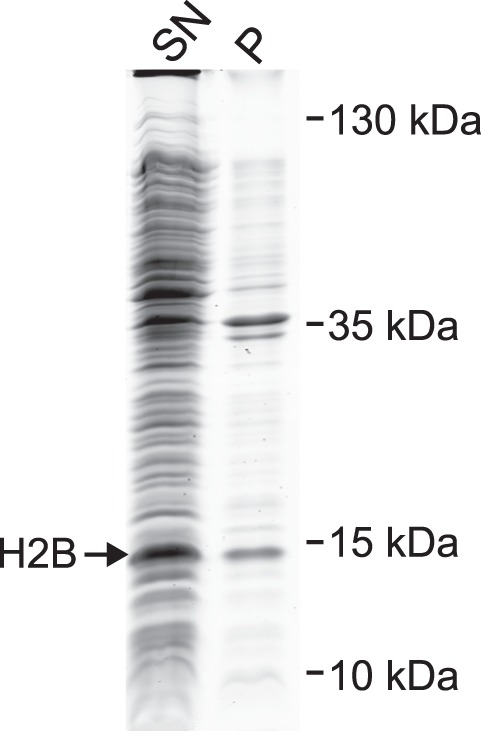
Histone extraction. Whole cell extracts were prepared under denaturing conditions from bacteria expressing *Drosophila* H2B by French Press and sonication. Cell debris and residual insoluble material were pelleted by centrifugation. Efficiency of the histone extraction was analyzed on Coomassie-stained SDS gels by loading equivalent amounts of the supernatant containing the solubilized histones (SN) and the corresponding pellet fraction (P). Most H2B was present in the supernatant.

To check the efficiency of the cell lysis and histone extraction, equivalent amounts of the pellet and supernatant fractions obtained after centrifugation (see step 2.3) were removed and proteins resolved by SDS-PAGE. Figure 2 shows exemplary results for histone H2B; similar results were obtained for the other three canonical histones. The majority of H2B was detected in the soluble extract, suggesting that cell lysis and histone extraction were efficient under the chosen conditions. Nevertheless, should a major fraction of the histone be found in the pellet, the extraction procedure should be repeated.

#### 2.3 Removal of cell debris by centrifugation

It is critical to remove most insoluble particles from the cell extract by centrifugation as they easily clog filters and chromatography media in the following steps. During centrifugation, a loose, viscous pellet may form. We therefore recommend recovering the soluble fraction by careful pipetting instead of decanting. If particles were accidentally carried over, a second round of centrifugation may be necessary.

### 3 Cation exchange chromatography and dialysis

**Figure 3:**
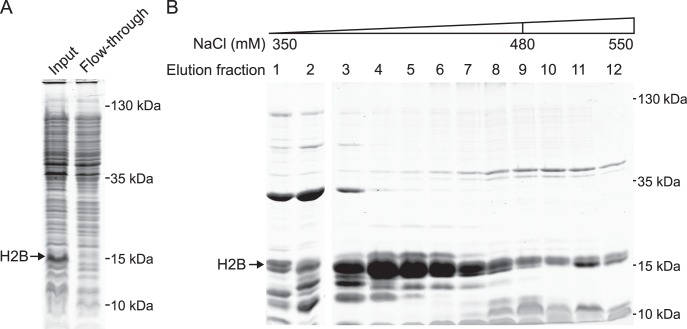
Histone purification by cation exchange chromatography. The whole cell extract from Figure 2 containing solubilized *Drosophila* H2B (SN) was filtered and applied to cation exchange chromatography under denaturing conditions. (A) Equivalent amounts of the filtered whole cell extract (Input) and the flow-through fraction of the cation exchange column were analyzed by SDS-PAGE. Most H2B bound to the chromatography resin. (B) H2B was eluted by a NaCl gradient as indicated. Fractions 4–8 were pooled and processed further as described in the main text.

The histones were purified from the cell extract by cation exchange chromatography under denaturing conditions. As the histones carry a net positive charge at the pH of the buffer (pH 5.2), they bound to the cation exchange resin at low salt concentrations, in contrast to the majority of bacterial proteins (Fig. 3A). The histones were then eluted by an increasing salt gradient.

#### 3.1 Filtering of the sample

Filtering of the supernatant from step 2.3 prior to the cation exchange is necessary to remove residual particulate material that can block the chromatography column. As conventional syringe filters easily clogged, we strongly recommend using syringe filters that contain a glass-fiber prefilter instead (HPF Millex; Millipore).

#### 3.2 Loading of the filtered sample onto a cation exchange column (HiTrap SP HP; 5 ml; GE Healthcare) pre-equilibrated in SAU 200 buffer

In a variation of the protocol, the sample is passed first over an anion exchange matrix that is attached on top of the cation exchange column to filter out impurities; see note in section 4.

#### 3.3 Washing of the cation exchange column with a minimum of 5 column volumes (CV) of SAU 200 buffer

For canonical histones from *Drosophila*, we recommend to continue washing with SAU buffer containing 250 mM NaCl (3 CV) and 300 mM NaCl (3 CV).

#### 3.4 Elution of the histones by applying a NaCl gradient

We recommend a gradient from SAU with 300 mM to 400 mM NaCl over 5 CV, followed by a gradient from SAU with 400 mM to 800 mM NaCl over 7 CV for all canonical *Drosophila* histones.

#### 3.5 Analysis of the protein content of the elution fractions by SDS-PAGE (Fig. 3B)

#### 3.6 Pooling of the fractions according to histone abundance and purity

As yields typically are not limiting, we suggest to pool according to purity.

#### 3.7 Dialysis of the pooled histone fractions against water

The pooled histone-containing fractions were extensively dialyzed against water to remove salts and urea.

### 4 Anion exchange filtration

By virtue of their positive charge, histones strongly bind to nucleic acids. Therefore, *E. coli-*derived nucleic acids may co-purify with histones. A contamination with nucleic acids can affect the concentration measurements of the purified histones (see below) or interfere with downstream applications. We therefore filtered the samples over an anion exchange column [7]–[9]. The negatively charged nucleic acids are expected to bind to the positively charged resin along with some contaminating proteins, whereas histones pass through the resin unimpededly.

Anion exchange filtration can be performed after the cation exchange chromatography according to steps 4.1 to 4.3. Alternatively, the filtration step is performed already prior to the cation exchange chromatography (see note in step 3.2), in which case steps 4.1 through 4.3 are omitted. In the latter variant, a HiTrap Q HP column is stacked on top of the HiTrap SP HP column. The cell extract from step 3.1 is then passed over the stack of two columns. Only one CV of SAU 200 is initially used to wash out the sample. The Q column is then detached from the FPLC system. Washing of the SP column resumes by applying the same wash protocol as above (step 3.3). The protein purities and the amount of contamination by nucleic acids (as judged by the absorption ratio at 260 and 280 nm) are comparable for both variants of the RHP protocol (Fig. S2 in File S1).

#### 4.1 Addition of Tris-Cl pH 8 to the dialysate to a final concentration of 15 mM

A buffered solution with a defined pH is necessary for robust binding of contaminations to the resin.

#### 4.2 Centrifugation and filtering of the sample to remove particles

Conventional syringe filters were used to filter the dialysate.

#### 4.3 Passing of the filtered sample over a HiTrap Q HP column

As expected, the histones were found in the flow-through of the anion exchange column.

### 5 Storage of purified histones

#### 5.1 Determination of the concentration

The concentration is most conveniently measured by absorption of UV light. It might be necessary to centrifuge the samples first to exclude insoluble particles. The extinction coefficients of *Drosophila* histones at 280 nm are listed in Table 1.

#### 5.2 Storage of the purified histones at –80°C until further use

Storage in aliquots that contain 0.5 to 2 mg is useful for most downstream applications.

### 6 Reconstitution of histone octamers

**Figure 4:**
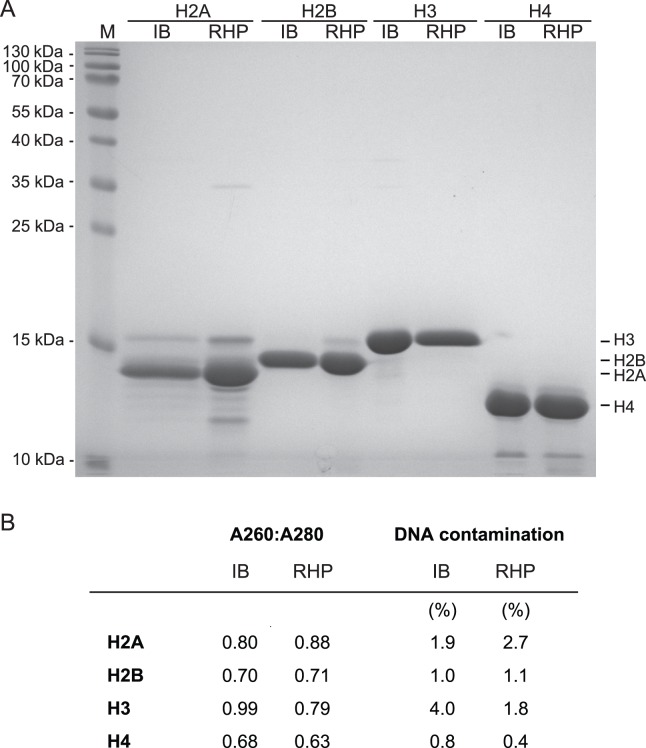
Side-by-side comparison of histone purities. Histones were purified according to the RHP protocol (RHP) and according to a published protocol that started with the preparation of inclusion bodies (IB; ref. 5). Both purification procedures started from the same amount of bacteria that were grown on the same day. (A) SDS-PAGE analysis. H2A showed the weakest overexpression (Fig. S1 in File S1) and is consequently the least pure. M: protein marker. (B) Purities.

All four canonical histones from *Drosophila* were purified according to the method outlined in steps 1–5 (Fig. 4). Next, we assembled histone octamers with these histone preparations essentially as described [2], [3]. Briefly, the four histones were lyophilized, dissolved in denaturing buffer and mixed. Dialysis was used to dilute the denaturant, allowing the histones to refold. Furthermore, the dialysis buffer contained 2 M NaCl, conditions that facilitate stable formation of histone octamers. Fully assembled histone octamers were separated from excess histones and contaminating bacterial proteins by size exclusion chromatography.

#### 6.1 Lyophilisation of each histone

#### 6.2 Dissolving of the lyophilized histones in guanidine hydrochloride-containing unfolding buffer to a concentration of 2–4 mg/ml

The histone suspensions were gently mixed at room temperature for 30 min. Note that histones should not remain in the unfolding buffer for an extended period of time (>3 h) [2], [3].

#### 6.3 Determination of the concentration of the histones by UV absorption as described in step 5.1

#### 6.4 Mixing of the histones

**Figure 5:**
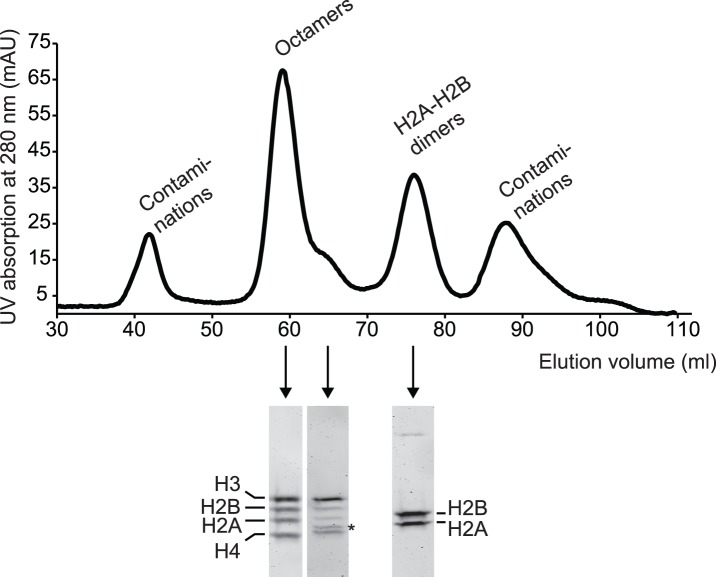
Histone octamer assembly. Histones purified according to the RHP protocol were assembled into octamers. The elution profile of the size exclusion chromatography column is depicted (upper panel). The protein content of selected elution fractions was analysed by SDS-PAGE (lower panel and Fig. S3 in File S1). Octamers eluted with a tailing shoulder, which contained a contaminating protein (asterisk).

We recommend mixing the histones for the assembly with a 1.2-fold excess of H2A and H2B over H3 and H4. Adding H2A and H2B in excess prevents the formation of free H3–H4 tetramers or histone hexamers, which are difficult to separate from histone octamers in the subsequent size exclusion chromatography step. Contrary, H2A–H2B dimers can be separated easily from the octamers (see step 6.7 and Fig. 5).

In addition to determining the concentration by UV absorption, we suggest analyzing the individual histone samples and the histone mix by SDS-PAGE and Coomassie-staining to judge the stoichiometry of the mix. If the ratios of the histones are unbalanced, the mix can be adjusted accordingly by addition of the underrepresented proteins before dialysis (see step 6.5).

#### 6.5 Dialysis into refolding buffer

#### 6.6 Removal of precipitates by centrifugation and filtration

#### 6.7 Size exclusion chromatography (Superdex 200)

Typically, four major peaks eluted from the column. SDS-PAGE analysis showed that the second and third peak contained the histone octamers and H2A–H2B dimers, respectively (Fig. 5 and S3 in File S1). The other peaks consisted of aggregates and low molecular weight impurities. Fractions containing pure octamers with the proper stoichiometry were pooled and stored as described in step 6.8.

We noticed that octamers occasionally eluted in a peak with a shoulder tailing towards later elution volumes. SDS-PAGE analysis revealed a contaminating band in these side fractions of the octamer peak (Fig. 5). This contamination presumably originated from a protein co-purifying with H4. We detected the contamination in H4 preparations irrespective of the purification method (Fig. 4 and data not shown). Additionally, late-eluting octamers are known to be contaminated by H3–H4 tetramers and histone hexamers, especially if H2A and H2B are limiting. It is therefore advisable to narrowly pool the peak.

#### 6.8 Storage of the octamers

The pooled fractions were concentrated, aliquots were shock-frozen in liquid N_2_ and stored at –80°C. Alternatively, the octamer sample can be stored at –20°C after addition of glycerol to a final concentration of 50% (v/v).

### Comparison of histone and octamer quality

**Figure 6:**
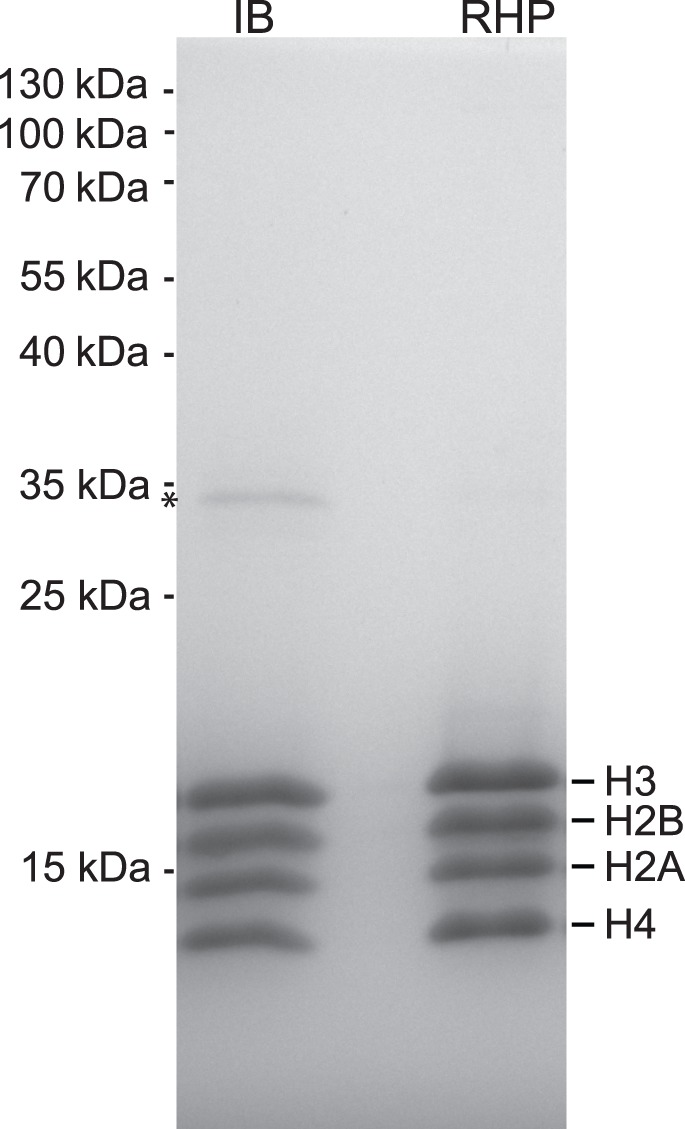
Quality control of the histone octamers. Stoichiometry and purity of the octamers assembled from the histones purified according to the RHP method outlined in the main text were analyzed by SDS-PAGE (RHP). An octamer preparation assembled from histones purified from inclusion bodies according to published protocols was loaded in parallel (IB; ref. 5). The asterisk marks a contamination that is present to a lesser extent in RHP octamers.

The RHP protocol presented above describes a straightforward way to purify histones. A direct comparison of the RHP method with our previous standard procedure [5] showed comparable purities for all four canonical histones (Fig. 4). Yields of the RHP method also fell within the expected range (2 to 15 mg per liter expression culture). Moreover, octamers assembled from histones purified by RHP or conventional inclusion body purification-based methods [5] had a similar purity and stoichiometry as judged by SDS-PAGE and Coomassie staining (Fig. 6). As expected, nucleosomes reconstituted with octamers that contained RHP histones were proper substrates for several nucleosome remodelling enzymes [11]–[13].

In summary, the RHP method offers a rapid and robust procedure to purify recombinant histones expressed in bacteria. RHP does not require laborious preparation of inclusion bodies and thus substantially reduces the required handling time. Two histone purifications can be readily started per day, such that all four histones are available within three days. So far, the protocol was successfully applied to prepare canonical *Drosophila* histones (this study), human histones (Jens Michaelis, personal communication), the *Drosophila* histone variant H2Av and various H2A, H3 and H4 mutants (data not shown). We expect that it will be useful also for the preparation of histones and histone variants from other organisms.

## Supporting Information

File S1 **Supporting Table S1 and Figures S1, S2 and S3.**
(DOCX)
