# Supplementary material for: Rapid Purification of Recombinant Histones
Source: PLoS One. 2014 Aug 4;9(8):e104029. doi: 10.1371/journal.pone.0104029 (PMC4121265; doi:10.1371/journal.pone.0104029)
Supplement: File S1 — Supporting Table S1 and Figures S1, S2 and S3. (DOCX) [file pone.0104029.s001.docx]

**Table S1:** DNA sequences of codon-optimized *Drosophila* H2A and H2B genes.

| **H2A** | ATGTCCGGCCGTGGGAAAGGCGGTAAAGTCAAGGGTAAGGCGAAGAGTCGCAGCAACCGCGCAGGTCTGCAATTTCCGGTGGGTCGCATTCATCGCCTGCTGCGTAAAGGTAACTACGCTGAACGCGTAGGCGCGGGCGCGCCTGTATATCTGGCTGCAGTCATGGAGTATCTGGCAGCCGAGGTTTTAGAACTGGCGGGCAACGCGGCTCGTGATAACAAAAAAACTCGTATCATCCCACGCCACCTGCAGCTGGCGATTCGCAATGACGAAGAATTAAATAAATTGCTGTCGGGCGTGACGATTGCCCAGGGCGGCGTTCTGCCGAATATCCAGGCCGTGTTGCTGCCGAAAAAAACCGAAAAAAAAGCCTAA |
| --- | --- |
| **H2B** | ATGCCACCGAAAACCTCCGGTAAAGCGGCCAAAAAAGCCGGCAAAGCCCAAAAGAACATCACGAAAACCGATAAGAAGAAGAAACGCAAACGCAAAGAGTCCTATGCGATTTACATCTATAAGGTGCTGAAACAGGTACATCCGGATACTGGcATTAGCAGTAAAGCCATGAGCATCATGAATAGCTTCGTGAATGACATCTTTGAACGCATTGCTGCAGAAGCGAGTCGTTTGGCTCACTACAACAAACGGTCGACCATTACCTCTCGTGAGATTCAGACTGCAGTTCGTCTGTTACTGCCTGGTGAACTCGCGAAACATGCGGTTTCAGAAGGCACAAAAGCAGTCACCAAATATACGTCGTCTAAATAA |


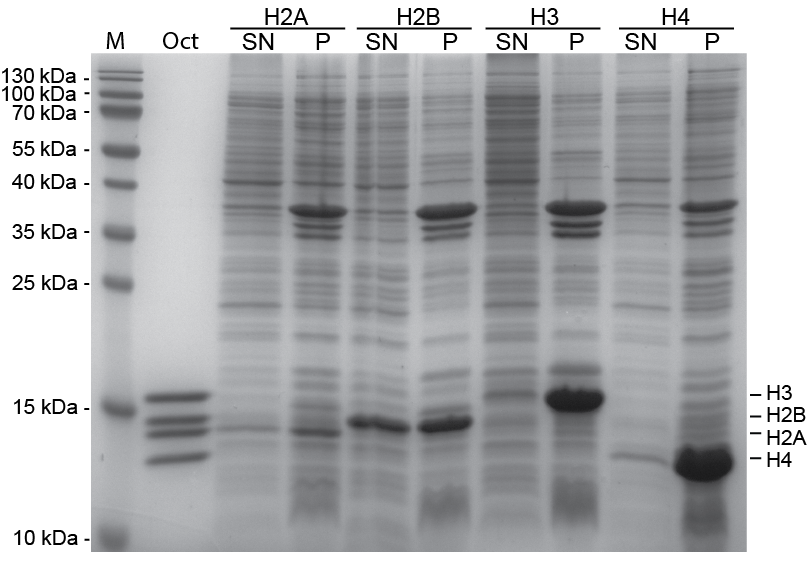


**Figure S1:** H2B does not fully partition into inclusion bodies. Whole cell extracts of cells expressing the indicated histones were prepared under non-denaturing conditions. Proteins in the soluble (SN) and insoluble (P) fractions of the whole cell extracts were analyzed by SDS-PAGE. Oct: histone octamer standard; M: protein marker.


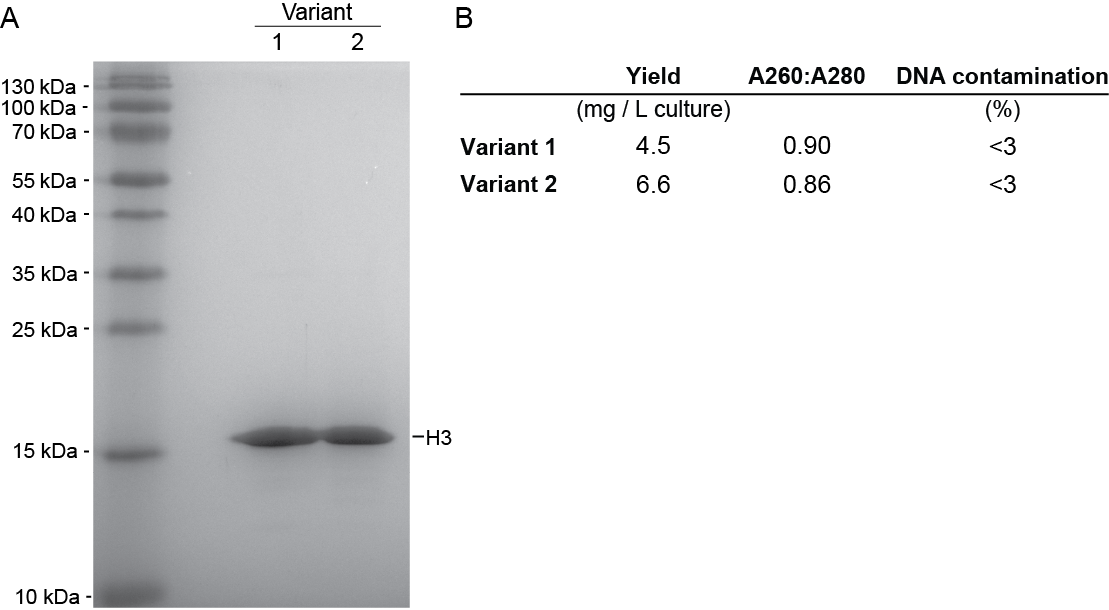


**Figure S2:** Comparison of two RHP variants. Histone H3-expressing cells were harvested and divided into two halves. H3 was purified according to the RHP protocol outlined in Figure 1B with the anion exchange filtration step succeeding cation exchange chromatography (Variant 1) or according to a variant in which anion exchange filtration and cation exchange chromatography were combined (Variant 2; see notes in Step 3.2). (A) SDS-PAGE of purified histone H3. (B) Purities and yields.

**
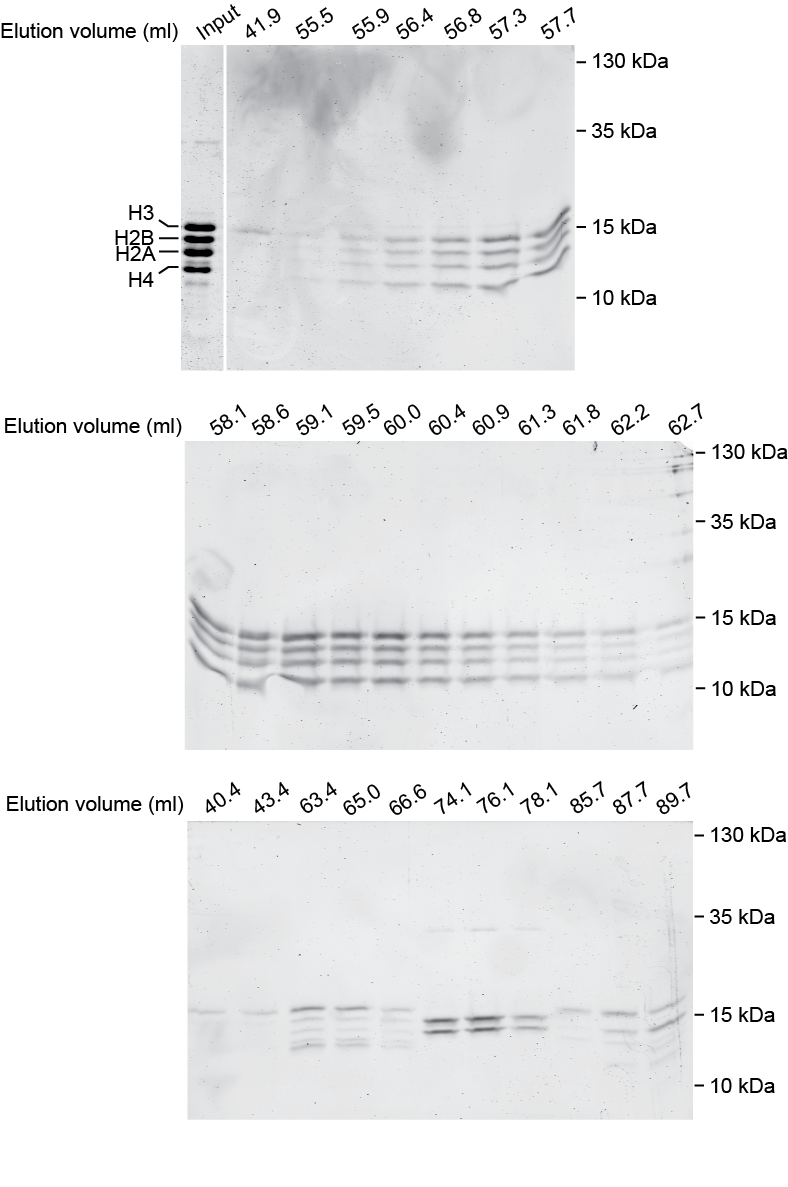
**

**Figure S3:** Purification of histone octamers by size exclusion chromatography. Analysis of the gel filtration elution fractions from the octamer purification shown in Figure 5. Selected elution fractions were analyzed by SDS-PAGE; the corresponding elution volumes are indicated.
